# Retention or sacrifice of posterior cruciate ligament has no effect on in vitro kinematics in medial pivot total knee arthroplasty

**DOI:** 10.1002/ksa.70121

**Published:** 2025-10-28

**Authors:** Johanna‐Maria Simon, Thomas Richard Niethammer, Christoph Thorwächter, Matthias Woiczinski, Felix Endres, Peter Ernst Müller, Boris Michael Holzapfel, Leandra Bauer

**Affiliations:** ^1^ Department of Orthopaedics and Trauma Surgery, Musculoskeletal University Center Munich (MUM), University Hospital Munich LMU Munich Munich Germany; ^2^ Experimental Orthopaedics, University Hospital Jena, Campus Eisenberg, Waldkliniken Eisenberg, Friedrich‐Schiller‐University Jena Jena Germany

**Keywords:** biomechanical study, knee kinematics, medial pivot, posterior cruciate ligament, total knee arthroplasty

## Abstract

**Purpose:**

The role of the posterior cruciate ligament remains debated, particularly in medial pivot designs intended to functionally substitute the posterior cruciate ligament. While some manufacturers recommend posterior cruciate ligament resection, evidence regarding its biomechanical relevance in medial pivot total knee arthroplasty is limited.

**Methods:**

This in vitro study evaluated knee kinematics in seven cadaveric knees implanted with a mechanically aligned medial pivot total knee arthroplasty (GMK Sphere). Each specimen was tested dynamically (30°–130° flexion) under three conditions: native knee, medial pivot with posterior cruciate ligament retained and with posterior cruciate ligament resected. Kinematic parameters (anterior‐posterior translation, tibial rotation, patellar shift and tilt) and quadriceps force were recorded using a 3D optoelectronic system in a validated dynamic knee rig.

**Results:**

Compared to native knees, medial pivot total knee arthroplasty increased anterior tibial translation and altered tibial rotation (*p* < 0.001), regardless of posterior cruciate ligament status. Patellar shift showed less medialization in deep flexion with posterior cruciate ligament retention in comparison to native conditions (*R* ^2^ = 0.20, *p* < 0.001). Quadriceps force differed from the native state but was unaffected by posterior cruciate ligament status; force patterns were primarily determined by the native knee (*R*² = 0.90, *p* < 0.001).

**Conclusion:**

Posterior cruciate ligament retention in medial pivot total knee arthroplasty does not significantly affect femorotibial or patellofemoral kinematics. The highly congruent medial design appears to reduce the functional importance of the posterior cruciate ligament. Therefore, intraoperative decisions regarding posterior cruciate ligament management may be made less on concerns about kinematic compromise.

**Level of evidence:**

N/A.

**Abbreviations**
AP: anterior‐posterior
CR: cruciate retaining
KA: kinematic alignment
MA: mechanical alignment
MP: medial pivot
NS: native state
PCL: posterior cruciate ligament
PE: polyethylene
PROM: patient‐reported outcome measure
PS: posterior stabilised
QF: quadriceps force
ROM: range of motion
TKA: total knee arthroplasty

## INTRODUCTION

In native knee joint kinematics the posterior cruciate ligament (PCL) functions as the primary restraint against posterior tibial translation, supporting femoral rollback during knee flexion, and also plays an important role in knee joint proprioception [14]. The commonly used cruciate retaining (CR) design in total knee arthroplasty (TKA) relies on an intact and functional PCL to counteract posterior drag forces from the hamstring muscles and facilitate femoral rollback [19]. A lack of femoral rollback can lead to tibiofemoral bony impingement beyond 90° of flexion [2]. However, due to the low conformity of CR components, an insufficient PCL may result in paradoxical anterior femoral translation and focal contact stress on the polyethylene (PE) insert [6]. Conversely, the posterior‐stabilised (PS) TKA design—developed to compensate for a deficient PCL in osteoarthritic knees—carries risks such as patellar‐clunk syndrome and condylar fractures [22].

The medial pivot (MP) design, based on the so called “ball and socket” principle medially and flat lateral surface as described by Pinskerova et al. [21] aims to functionally substitute for the PCL. Its high anterior lip is intended to prevent posterior tibial subluxation [23]. Implant manufacturers currently recommend PCL resection when using an MP design to avoid abnormal kinematics (as stated in the Medacta instructions manual). PCL resection is also commonly used to reduce flexion gap tightness in TKA. Nonetheless, suggested benefits of PCL retention include enhanced proprioception, preservation of near‐natural kinematics, fewer patella‐related complications and improved quadriceps strength [18, 20].

To date, only two studies have investigated the influence of the PCL in MP TKA. Bae et al. compared clinical outcomes in patients receiving a mechanically aligned (MA) MP TKA with either the PCL retained or resected [1], finding no significant differences in postoperative range of motion (ROM) and patient‐reported outcome measures (Knee Society Score). In contrast, Nedopil et al. observed a loss of internal tibial rotation during flexion in kinematically aligned MP TKA following PCL resection, based on cadaveric testing, compared to both PCL‐retained and native knee joint kinematics [16]. A loss of internal tibial rotation may correlate with increased patellar tilt, lateral displacement, and anterior knee pain [9, 15].

Biomechanical data specifically addressing the influence of the PCL on knee kinematics in MP TKA remain limited. Yet, such data are essential, as clinical studies often cannot adequately capture detailed tibiofemoral and patellofemoral motion patterns. Dynamic, weight‐bearing simulations using knee rigs help bridge this gap by replicating physiological loading conditions and partially approximating clinical behaviour.

Therefore, the aim of this study was to investigate the biomechanical role of the PCL in MP‐TKA focusing on potential differences in tibiofemoral and patellofemoral kinematics as well as patellofemoral using native knee kinematics as reference. We hypothesised that PCL preservation in MP TKA would not result in significant differences in tibiofemoral and patellofemoral kinematics compared to PCL resection.

## MATERIALS AND METHODS

### Specimen and implantation

Ethics approval for this study was granted by the Ethics Committee of the University of Munich (#20‐829). Seven fresh‐frozen human cadaver specimens (including femoral head and upper ankle joint) were used for biomechanical testing. The sample included three male and four female donors (four right, three left legs) with a mean age of 81 ± 6 years. Specimens were stored at −20° and thawed at room temperature 24 h prior to testing. All specimens exhibited a neutral mechanical leg axis (0 ± 3°) without relevant signs of osteoarthritis, assessed via X‐ray fluoroscopy. The femoral and tibial shafts were shortened (22 and 20 cm, respectively) and all skin and subcutaneous tissue were removed. Muscles were dissected, and the tendons of the quadriceps femoris, biceps femoris and semitendinosus muscles fixed in finger clamps using FibreWire sutures (Arthrex, Naples, USA). The fibular head was fixed to the tibia using a cortical screw to ensure stability during testing. Both tibial and femoral ends of the specimens were embedded in metal pots using epoxy resin and mounted into the dynamic knee rig. To avoid malalignment of the femoral component, the posterior femoral condyles were aligned parallel to the hip flexion axis in the transverse plane.

**Figure 1:**
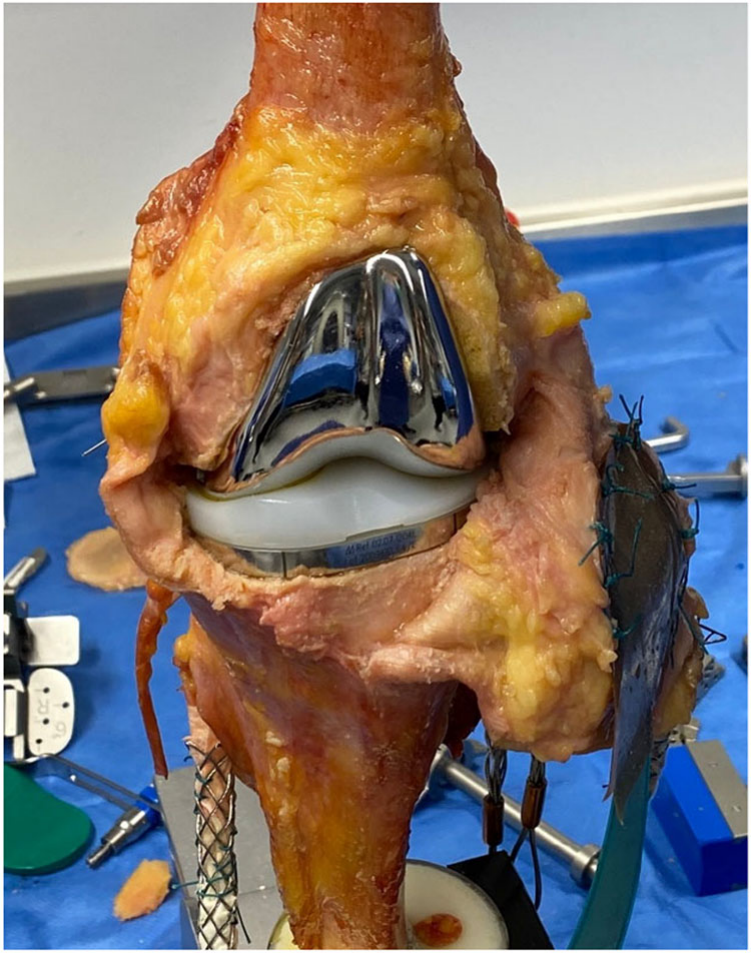
Human cadaver specimen with a medial pivot (MP) total knee system (GMK Sphere, Medacta International, Castel San Pietro, Switzerland).

A MP total knee system (GMK Sphere, Medacta International, Castel San Pietro, Switzerland) was used for implantation (Figure 1). The procedure was performed by the first author, an experienced knee surgeon, following a MA technique with 3° external femoral component rotation and a 3° posterior tibial slope. Ligament balancing was performed to achieve symmetric gaps, ensuring stability in extension and adequate flexion. The PCL was initially preserved and protected by carefully chiselling around it prior to tibial resection. The patella was not resurfaced but was mildly reshaped, and osteophytes were removed if necessary. A 10‐mm PE insert was used in all specimens. Correct implant positioning was confirmed via X‐ray fluoroscopy. Following initial kinematic testing, the PCL was resected without altering the implant configuration.

### Biomechanical setup

**Figure 2:**
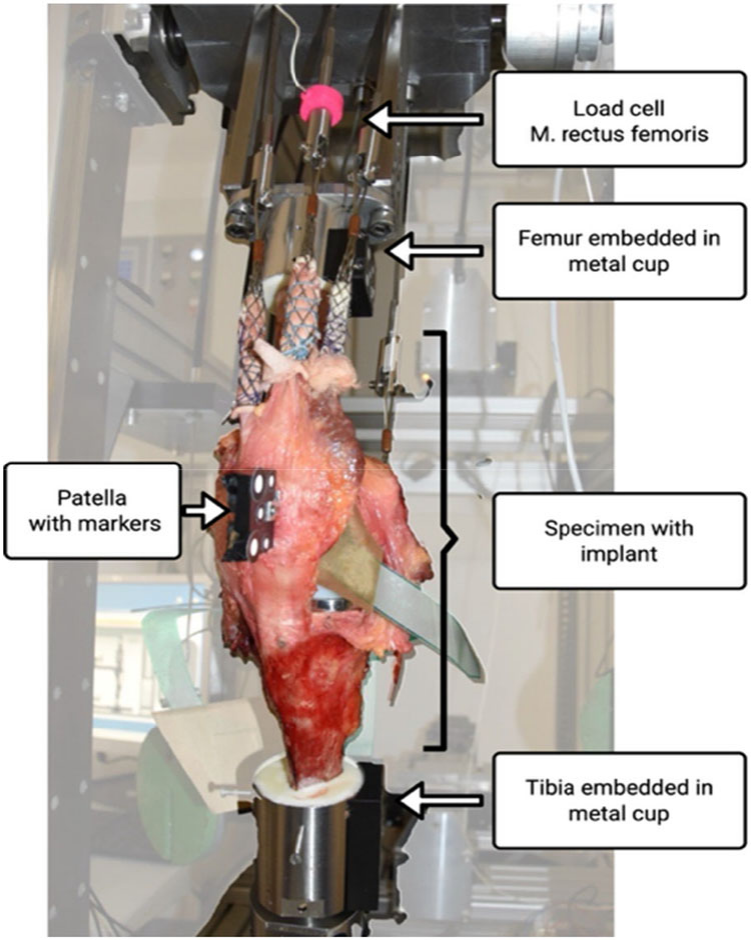
Biomechanical setup with the human knee joint specimen mounted onto the knee rig.

Kinematic measurements were performed using a validated dynamic knee rig (Figure 2), which provides six degrees of freedom, as previously described [3, 4, 25]. The knee rig allows for active knee flexion under load with a flexion range from 30° to 130°, while maintaining a constant ground reaction force (GRF, 50 N). The ground reaction force was applied via a sensor (84176002 Burster, Gernsbach, Germany) connected to the rectus femoris tendon. To simulate muscle activity, 2 kg weights were attached to the vastus medialis, vastus lateralis, semitendinosus and biceps femoris tendons. Real‐time movement control was achieved using a custom LabView programme (version 8.6, National Instruments). A 3D optoelectronic measurement system (ARAMIS 3D Camera 2.3 M, GOM GmbH, resolution 1936 × 1216 pixels, measurement volume 1230 × 790 × 790 mm, accuracy 24.6 μm in the focal plane and 49.2 μm outside the focal plane) recorded motion using reflective markers placed on the femur, tibia and patella.

Initial measurements were conducted in the native knee condition, followed by measurements after TKA with PCL preservation, and finally after PCL resection.

### Data analysis and statistics

Data from the optoelectronic system were synchronised and interpolated with corresponding flexion angles from the knee rig, using an established method [10, 26]. The subsequent data processing was conducted using a custom MATLAB script (Version 24.2, 2024b, The MathWorks Inc, Natick, MA, USA).

Anterior‐posterior (AP) translation was analysed as global translation of the distal femur—defined as movement of the midpoint between the femoral condyles relative to the tibia. Femorotibial kinematics were described using a fixed‐femur approach to illustrate tibial movement. For patellofemoral kinematics, patellar shift was defined as mediolateral translation relative to the femur, and patellar tilt as internal and external rotation. Quadriceps force required to maintain a constant GRF was directly measured at the quadriceps tendon during flexion, with results reported as means and standard deviations. Translation/rotation at 30° flexion was set as the baseline for all kinematic analyses. Kinematic calculations followed the methods of Bull et al. and Grood and Suntay [5, 12]. Results are represented as mean values across all seven specimens, with 95% confidence intervals. Statistical analyses were conducted using RStudio software (R version 4.3.1, RStudio, Inc., Boston, MA, US). Functional regressions (pffr) were calculated using the *refund* package (version 0.1‐35) [11]. The native knee served as the reference condition to evaluate the effect of PCL preservation or resection on the outcome parameters: quadriceps force, femorotibial and patellofemoral kinematics during flexion. Significance level was set at *p* = 0.05.

## RESULTS

### Femorotibial kinematics

**Figure 3:**
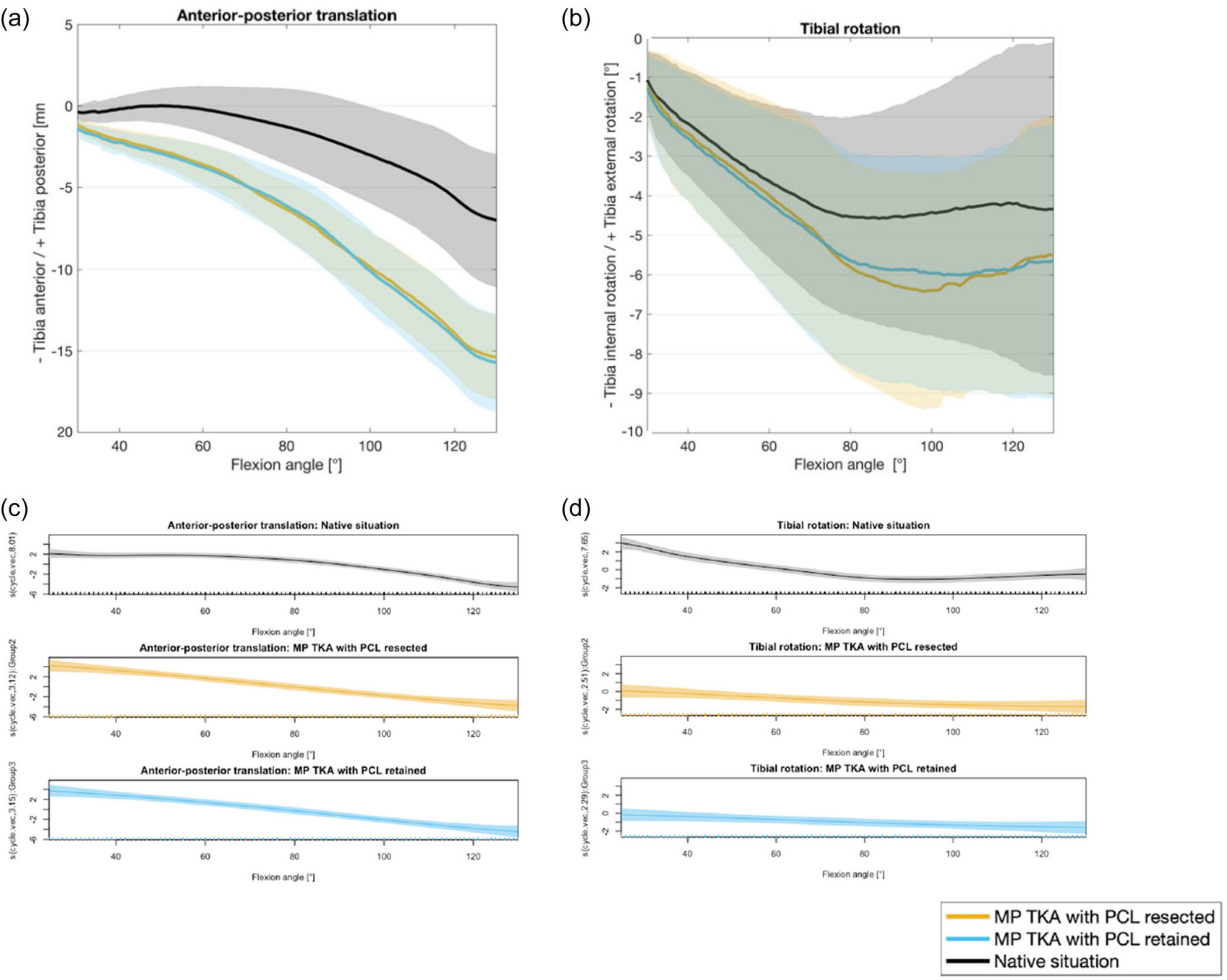
Femorotibial kinematics with mean and 95% confidence interval for (a). AP translation, (b) tibial rotation with MP TKA with PCL resected (orange), MP TKA with PCL retained (blue) and native situation (black); functional regression analysis for (c). AP translation and (d) tibial rotation. AP, anterior‐posterior; MP, medial pivot; PCL, posterior cruciate ligament; TKA, total knee arthroplasty.

Analysis of AP translation revealed increased anterior tibial displacement following TKA compared to the native knee across the entire flexion range (30°–130°). Notably, no significant difference in AP translation was observed between TKA conditions with preserved or resected PCL (Figure 3a). This finding was confirmed by functional regression analysis (*R* ^2^ = 0.47, *p* < 0.001) (Figure 3c).

Regarding tibial rotation, both native and TKA knees exhibited progressive internal tibial rotation up to approximately 70° of flexion (Figure 3b). In TKA knees, internal rotation continued slightly further (up to ~80°), followed by a plateau extending to 130°. Functional regression analysis demonstrated that tibial rotation was primarily influenced by the native knee kinematics up to 70° of flexion (Figure 3d). Although TKA significantly affected tibial rotation up to 80°, there was no difference between PCL‐retained and PCL‐resected conditions (*R* ^2^ = 0.19, *p* < 0.001).

### Patellofemoral kinematics

**Figure 4:**
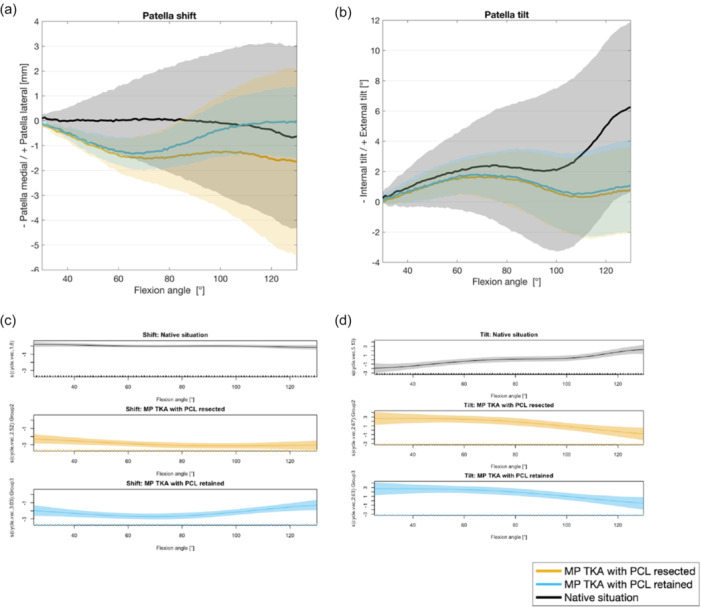
Patellofemoral kinematics with mean and 95% confidence interval for (a) patellar shift, (b) patellar tilt with MP TKA with PCL resected (orange), MP TKA with PCL retained (blue) and native situation (black); functional regression analysis for (c) patellar shift and (d) patellar tilt. AP translation and (d) tibial rotation. AP, anterior‐posterior; MP, medial pivot; PCL, posterior cruciate ligament; TKA, total knee arthroplasty.

Analysis of patellar shift showed no average displacement in the native knee; however, wide confidence intervals indicated high inter‐specimen variability (Figure 4a). After TKA, a medial patellar shift was observed up to 60° flexion. With retained PCL, the patella gradually returned toward a central position with increasing flexion. In contrast, with a resected PCL, the patella remained medially displaced without further shift at deeper flexion angles. Functional regression analysis confirmed a moderate influence of the PCL status in deep flexion (*R* ^2^ = 0.20, *p* < 0.001) (Figure 4c).

For patella tilt, both native and TKA knees showed external patellar rotation up to 70° flexion (Figure 4b). Beyond this, the native knees exhibited further external tilt, whereas TKA knees showed no additional change. There was no significant difference between PCL conditions. Functional regression analysis indicated that patellar tilt was primarily influenced by native kinematics, with TKA effects most pronounced at high flexion angles (*R* ^2^ = 0.03, *p* < 0.001) (Figure 4d).

### Knee joint loading

**Figure 5:**
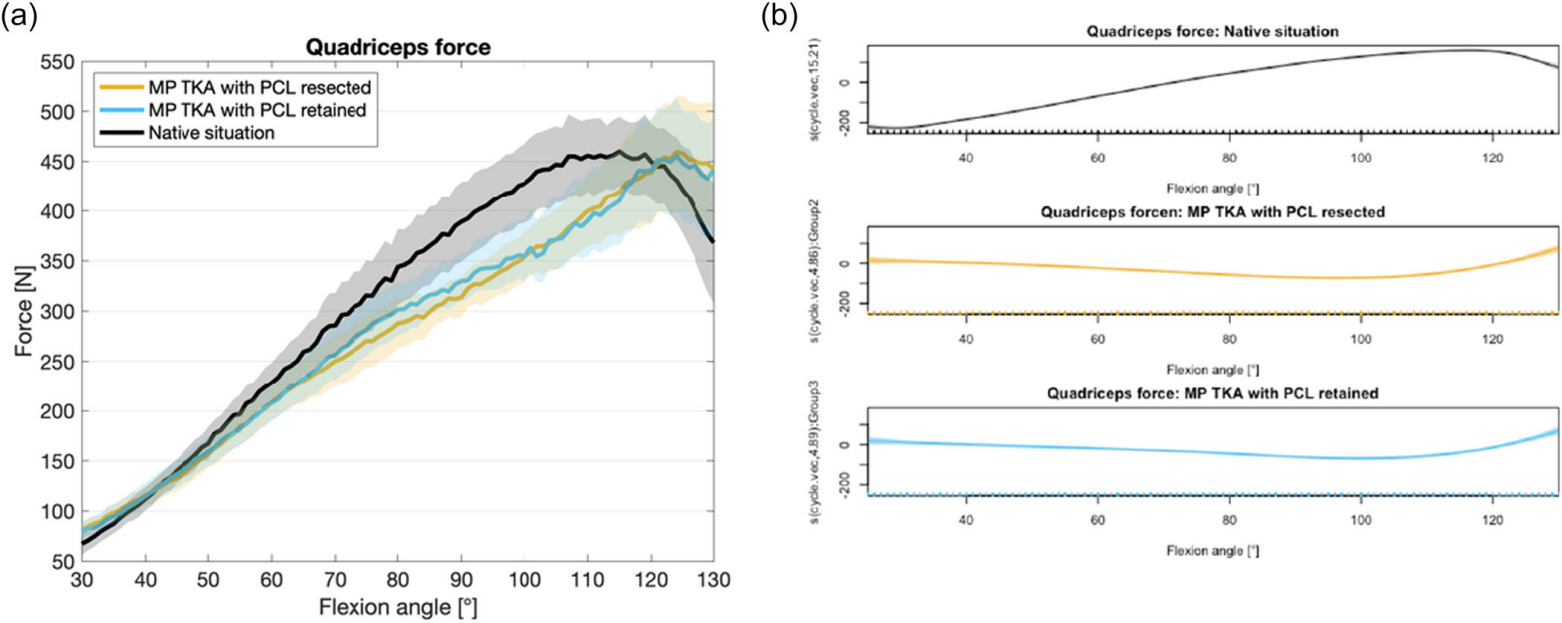
(a) Quadriceps force with mean and 95% confidence interval with MP TKA with PCL resected (orange), MP TKA with PCL retained (blue) and native situation (black); (b) functional regression analysis for quadriceps force. AP, anterior‐posterior; MP, medial pivot; PCL, posterior cruciate ligament; TKA, total knee arthroplasty.

Quadriceps force (QF) increased with knee flexion up to approximately 110°, followed by a plateau between 110° and 130° (peak values: native: 460 N [CI 423– 497 N], PCL resected 459 N [CI 399–519 N], PCL retained 456 N [CI 395–517 N]). In TKA knees, the rate of QF increase decreased from approximately 70° flexion, with continuous increase until 120°, and a plateau thereafter (Figure 5a). Functional regression revealed that QF was predominantly influenced by native kinematics up to 100° flexion, with significant effects of TKA from 80° onward (*R* ^2^ = 0.90, *p* < 0.001) (Figure 5b). However, this difference diminished towards deeper flexion angles. PCL status had no effect on QF.

## DISCUSSION

This study investigated tibiofemoral and patellofemoral kinematics, as well as quadriceps force, comparing native knees to MP‐TKA with preserved or resected PCL. Femorotibial kinematics (AP translation, tibial rotation) and quadriceps force were not significantly affected by PCL status. Patellofemoral kinematics showed slight medialization of the patella in high flexion with PCL retention, though without statistical significance.

Typically, changes in patellar tracking are associated with altered tibial rotation or femoral component positioning. Since both were unchanged in this study, the minor medial shift observed may be attributed to inter‐individual anatomical variability and differing levels of patellar laxity.

Contrary to the findings by Nedopil et al. [16], who reported increased internal tibial rotation with PCL retention in kinematically aligned (KA) MP‐TKA using in vivo goniometric assessment, our in vitro knee rig study found no such effect. The differing alignment strategies (KA vs. MA) and methodological differences likely account for these discrepancies. KA has been associated with more native‐like internal tibial rotation and reduced quadriceps force [4, 8], particularly when combined with MP implants rather than CR designs [3, 25]. Therefore, in terms of in‐vitro biomechanical aspects, PCL retention appears to be advantageous in KA MP‐TKA.

Elorza et al. also demonstrated increased internal tibial rotation in MP‐TKA compared to CR‐TKA under KA, using 3D/2D image registration, though no differences in PROMs were observed [7]. They included 25 patients with bilateral TKA comparing MP‐TKA and CR‐TJKA with PCL retention.

A recent review suggests that combining MP‐TKA with KA results in kinematics closer to the native knee than MA with traditional designs [13]. However, as shown in our study, the biomechanical advantages of PCL retention in MA MP‐TKA remain unproven.

Our findings align with those of Bae et al. [1], who reported no differences in ROM or PROMS between PCL‐retained and PCL‐resected MP‐TKA under MA. Similarly, Sathappan et al. [24] examined 114 ultra‐congruent TKAs with either PCL retention or resection, depending on PCL tightness. They found no clinical differences between groups with or without PCL resection.

These studies support the notion that the theoretical concern of kinematic conflict in congruent inserts due to retained PCL is not clinically relevant. The high congruency of MP inserts appears to render the PCL functionally redundant.

Although PS designs in TKA were developed to address PCL insufficiency, evidence does not clearly support replacing the PCL with a cam‐post mechanism over a congruent insert [24]. Given the increased risk of patellar clunk syndrome and condylar fractures associated with PS designs [22], MP‐TKA may be a preferable alternative, even in PCL‐deficient knees.

Taking these findings into account, it may be deduced that the use of a MP implant in TKA is possible, regardless of the status of the PCL, and should be followed by intraoperative decision making on PCL resection or PCL retention depending on flexion gap tightness, intraoperative ROM (and anterior lift‐off of the trial insert).

Preserving the PCL can lead to better proprioception and a more natural feeling in the knee during active movement, as well as lower shear forces on the implants [18, 20]. However, when finite element analyses are taken into account, PCL tension can play an important role, as a tight PCL has been shown to cause greater femoral rollback compared to a loose PCL, at the expense of a narrower flexion gap, higher joint compression, and potential PE wear [17, 27].

The presented study has a few limitations. Firstly, it is an in vitro study with a small sample size (*n* = 7), which makes it difficult to directly transfer the findings to the in vivo situation. However, power analysis indicated that six specimens would suffice. Given the ethical and logistical challenges of cadaveric studies, using a dynamic knee rig, samples sizes of 7–10 are standard in this research field. The investigation of biomechanical aspects such as kinematics and pressure loads within the tibiofemoral and patellofemoral joints cannot be evaluated in clinical studies but provides important insights for improving implant design and alignment. The Munich knee rig, with its simulation of active knee flexion controlled by the rectus femoris muscle, is an excellent option for simulating the kinematics and loads of the knee joint. To ensure comparability, healthy knee joint specimens that showed no severe signs of osteoarthritis were used. However, it is known that the PCL is often attenuated in osteoarthritis, whereas the PCLs in our specimens were rated competent. Nevertheless, despite the best possible protective measures for the PCL during implantation, damage may have occurred which, together with the average age of the donors (81 years) and the associated possibility of increased ligament laxity, may have led to a reduction in kinematic differences.

Due to inconsistencies of the femoral component in medial constrained implant designs, these kinematic findings can only be transferred to true medial pivot/ball‐in‐socket designs.

## CONCLUSION

In this in vitro study, PCL retention in MA MP‐TKA did not result in significant differences in tibiofemoral or patellofemoral kinematics. Intraoperative decisions on PCL management may therefore be guided by individual factors such as flexion gap balance and implant stability, as the highly congruent medial design of MP implants appears to diminish the functional importance of the PCL. Further studies, including gait analyses of larger patient cohorts and comparisons of KA versus MA strategies, are warranted to more precisely define the role of the PCL in MP‐TKA.

## AUTHOR CONTRIBUTIONS

All authors contributed to the study conception and design. Material preparation, data collection and analysis were performed by Johanna‐Maria Simon, Leandra Bauer and Christoph Thorwächter. The first draft of the manuscript was written by Johanna‐Maria Simon and Leandra Bauer and all authors commented on previous versions of the manuscript. All authors read and approved the final manuscript.

## CONFLICT OF INTEREST STATEMENT

P. E. Müller is a consultant for the Medacta shoulder system and B. Braun Aesculap. This in no way influenced the results of this study. The authors declare no conflict of interest.

## ETHICS STATEMENT

The study was conducted according to the guidelines of the Declaration of Helsinki. The in vitro study was approved by the Ethics Committee of the University of Munich (#20‐829).

## Data Availability

The data that support the findings of this study are available from the corresponding author upon reasonable request.
